# Inhibition of esophageal cancer progression through HACE1-TRIP12 interaction and associated RAC1 ubiquitination and degradation

**DOI:** 10.7150/jca.93833

**Published:** 2024-04-08

**Authors:** Ya Hu, Ziyi Zhu, Yanhua Xu, Muhammad Fakhar Zaman, Yuxuan Ge, Jinming Hu, Xi Tang

**Affiliations:** 1 Health Science Center, Yangtze University, Jingzhou City, Hubei Province, China, 434023.; 2 Department of Oncology, Jingzhou Central Hospital, Jingzhou City, Hubei Province, China, 434020.

**Keywords:** esophageal cancer, HACE1, RAC1, ubiquitination, protein-protein interaction

## Abstract

**Objective:** This study investigated the significance of HECT domain and ankyrin repeat containing E3 ubiquitin protein ligase 1 (HACE1) in esophageal cancer (ESCA) and its underlying mechanism in ESCA regulation through the induction of RAC1 ubiquitination and degradation.

**Methods:** Characterization studies of HACE1 in ESCA clinical tissues and cell lines were performed. Next, the effects of HACE1 on the biological behavior of ESCA cells were examined by silencing and overexpressing HACE1. Protein-protein interactions (PPIs) involving HACE1 were analyzed using data from the String website. The function of HACE1 in RAC1 protein ubiquitination was validated using the proteasome inhibitor MG132. The effects of HACE1 on ESCA cells through RAC1 were elucidated by applying the RAC1 inhibitor EHop-016 in a tumor-bearing nude mouse model. To establish the relationship between HACE1 and TRIP12, rescue experiments were conducted, mainly to evaluate the effect of TRIP12 silencing on HACE1-mediated RAC1 regulation in vitro and in vivo. The PPI between HACE1 and TRIP12 and their subcellular localization were further characterized through co-immunoprecipitation and immunofluorescence staining assays, respectively.

**Results:** HACE1 protein expression was notably diminished in ESCA cells but upregulated in normal tissues. HACE1 overexpression inhibited the malignant biological behavior of ESCA cells, leading to restrained tumor growth in mice. This effect was coupled with the promotion of RAC1 protein ubiquitination and subsequent degradation. Conversely, silencing HACE1 exhibited contrasting results. PPI existed between HACE1 and TRIP12, compounded by their similar subcellular localization. Intriguingly, TRIP12 inhibition blocked HACE1-driven RAC1 ubiquitination and mitigated the inhibitory effects of HACE1 on ESCA cells, alleviating tumor growth in the tumor-bearing nude mouse model.

**Conclusion:** HACE1 expression was downregulated in ESCA cells, suggesting that it curbs ESCA progression by inducing RAC1 protein degradation through TRIP12-mediated ubiquitination.

## Introduction

Esophageal cancer (ESCA) has been one of the most aggressive of all gastrointestinal malignancies. A global statistical report reveals 604,100 new cases of ESCA and 544,076 associated deaths in 2020, accounting for, respectively, 3.1% and 5.5% of all malignant tumors 1. Among 36 different cancers, ESCA ranks tenth in terms of the incidence rate of newly diagnosed cases and sixth in terms of the mortality rate 1. Surgical interventions, combined with radiotherapy or chemotherapy, have demonstrated effectiveness in reducing ESCA tumor burden and extending survival 2-3. Despite these treatment efforts, ESCA has a poor prognosis: at the time of diagnosis, 50% of patients are already in the advanced stage, the 5-year survival rate is only approximately 20%, and the local recurrence rate is as high as 68%-84% 4-5. Thus, a comprehensive investigation into the underlying mechanisms of ESCA holds immense value for both diagnosis and treatment.

HECT domain and ankyrin repeat containing E3 ubiquitin protein ligase 1 (HACE1) is an E3 ubiquitin ligase consisting of a HECT domain 6. Apart from its role in maintaining nervous system homeostasis and cardiac function 7-8, it acts as a tumor suppressor. For instance, HACE1 deficiency increases the proliferative and metastatic capabilities of liver cancer cells and is associated with malignant pathological features and poor patient survival rates 9 Moreover, HACE1 exerts tumor-suppressive effects in digestive tract malignancies such as colorectal cancer 10 and gastric cancer 11. Nonetheless, the precise clinical features and regulatory mechanisms of HACE1 in ESCA remain unclear.

The RAC1 protein, a GTPase, orchestrates diverse cellular functions such as apoptosis, secretion, adhesion, and motility by modulating several downstream effector proteins 12-14. In ESCA, RAC1 fosters cell proliferation, metastasis, and drug resistance. Consequently, the inhibition of RAC1 is considered a promising strategy for ESCA treatment 15-16. HACE1, which is a post-transcriptional regulator of RAC1, governs RAC1 activity by inducing its ubiquitination and subsequent degradation 7, a phenomenon observed in Parkinson's disease. Reduced HACE1 levels elevate RAC1 protein abundance, promoting breast cancer metastasis 17. These observations suggest the potential involvement of HACE1 in ESCA progression through its interaction with RAC1.

This study investigated the characteristic features of HACE1 in ESCA by analyzing whether HACE1 regulates the biological behavior of ESCA cells through RAC1 and exploring the mechanisms underlying RAC1 ubiquitination at the protein level.

## Materials and methods

### ESCA tissues

Twenty pairs of ESCA tissues and adjacent normal tissues were obtained from the pathology department. The samples were formalin-fixed, paraffin-embedded esophageal cancer specimens collected during diagnostic endoscopy from patients seeking treatment at Jingzhou Central Hospital. Adjacent normal tissues were taken from normal appearing esophageal mucosa greater than 5 cm proximal to tumor margin and confirmed to be 100% normal by histopathological examination. All patients provided informed consent during sampling. The patient group included 6 patients with esophageal adenocarcinoma and 14 patients with esophageal squamous cell carcinoma, 12 males and 8 females with an average age of 56.8 years. The Ethics Committee of Jingzhou Central Hospital reviewed our study and gave its approval to this research (No.2023-033-01). All procedures in the study were performed in conformity with the Declaration of Helsinki.

### Cell culture

Healthy human esophageal epithelial cells (HEECs) and ESCA cells (OE19, KYSE-30, TE-1, and Eca109) were purchased from American Type Culture Collection and maintained in Dulbecco's modified Eagle's medium (DMEM; Gibco, USA), supplemented with 10% fetal bovine serum, 100 mg/mL of streptomycin, and 100 U/mL of penicillin (Sigma-Aldrich, USA). The cells were cultured in a 5% CO_2_ incubator at 37 °C and 95% humidity. To inhibit the degradation process occurring after ubiquitination, MG132 was used to block the proteasome system. MG132 (final concentration: 20 μmol/L) was added to the cell culture plate, and the cells were cultured for 24 h. Cell viability was measured using the Cell Counting Kit-8 assay kit (Beyotime, Shanghai, China), following the manufacturer's instructions.

### Cell transfection

Eca-109 cells were used for cell transfection assays to overexpress HACE1, and TE-1 cells with relatively high HACE1 expression were used for cell transfection assays to silence HACE1. siHACE1, HACE1, siTRIP12, β-catenin, and the corresponding Negative control (NC) plasmids were purchased from GenePharma Co., Ltd. (China). The plasmid (50 pmol; 0.67 μg) was diluted with 25 μL of serum-free DMEM, forming reagent A. Entranster^™^-R4000 (Engreen; 1 μL) was mixed 24 μL of serum-free DMEM for 25 min, forming reagent B. Reagent A (25 μL) and reagent B (25 μL) were mixed thoroughly (aspirate 10 times using the pipette), after which the solution was allowed to stand for 15 min, forming the transfection complex. Cells were plated (with 0.45 mL of complete medium per well) and were transfected with 50 μL of transfection complex. The NC plasmid was used as the control.

### Xenotransplantation assay

Four-week-old male athymic BALB/c nude mice (Weitonglihua. Co., Ltd, China) were used for cell tumorigenesis assay. The animals were housed and raised under conditions of 24±1 ℃ temperature and 60±5% relative humidity. The mice were allocated to different groups (NC, HACE1, HACE1+siTRIP12) using the random number table method. Cells (5 × 10^6^) were resuspended in 200 μL of phosphate-buffered saline (PBS) and injected into the armpit area of the nude mice. Twenty-eight days post-transplantation, the mice received euthanasia by i.p. injection of pentobarbital sodium (250 mg/kg), especially after confirming that the tumor diameter was greater than 2 cm (not involved in this experiment), and the tumors were collected and weighed. All animal protocols in this study were approved by the Ethical Committee of Health Science Center, Yangtze University (No. YZLL2023-02).

### Immunohistochemical staining

After the paraffin-embedded sections were deparaffinized and washed with 3% hydrogen peroxide (for 5-10 min at room temperature) and PBS, the samples were incubated at room temperature for 10 min. Immunohistochemical staining was performed with rabbit polyclonal anti-HACE1 antibodies (1:50 dilution, ab237045; Abcam, USA) and biotin-labeled secondary antibodies. DAB was used for color development. The staining tissues were observed under a microscope (Olympus, Japan).

### qRT-PCR assay

The cells were homogenized, after which 600 μL of TRIzol™ Reagent (Thermo Fisher Scientific, USA) was added, and the cells were mixed with this reagent for 5 min. Isopropanol was then added to precipitate the total RNA in the aqueous phase. The isolated RNA was reverse transcribed into cDNA using PrimeScript™ RT Reagent Kit (RR047A; Takara). Amplification was performed Quantitative reverse transcription-polymerase chain reaction (PCR) with SYBR Green Reagent (Takara, Japan). The reaction conditions for the PCR assay were as follows: 94 °C for 3 min, 40 cycles of 94 °C for 15 s, 58 °C for 20 s, and 72 °C for 30 s. mRNA expression was normalized to GAPDH using the 2^-ΔΔCt^ method. The primers used for this assay were as follows: HACE1 F: 5′-CTCGCAGCTCAGTGCTCTA-3′, R: 5′-TTACTGGGGGAGGATGGACC-3′; TRIP12 F: 5′-ATGACTCGAGCTCAGAGGGT-3′, R: 5′-AGGTAGGGGTCGCTGGG-3'; GAPDH F: 5′-TTTCTGACTCCGTGAACCGC-3′, R: 5′-AGTCCTTCCACGATACCAAAGTT-3′.

### Western blot

Western blotting was performed according to the standard procedure. The following primary antibodies were used: anti-HACE1 (ab133637), anti-TRIP12 (ab86220), anti-RAC1 (ab97732), anti-CCND1 (ab226977), anti-Bcl-2 (ab182858), anti-MMP2 (ab181286), anti-GAPDH (ab181602). The blotted membrane was washed twice with Tris-buffered saline/0.1% Tween 20 (TBST) solution. The membrane was then incubated with secondary antibodies (1:2000 dilution, ab6721) for 2 h at 37 °C. The membrane was washed thrice with TBST. The protein immunoblots were visualized using an enhanced chemiluminescence (ECL) Kit (Solarbio), and proteins were detected using IPP6.0 (Media Cybernetics, USA).

### Ubiquitination assay

Cells were incubated with radioimmunoprecipitation assay lysis solution on ice for 30 min. Total protein was extracted by centrifugation (4 °C, 12000 × *g*, for 20 min). RAC1 protein was isolated by immunomagnetic beads (Miltenyi Biotec, Germany). Subsequently, western blotting, sodium dodecyl sulfate-polyacrylamide gel electrophoresis, and ubiquitinated antibody (Sigma, USA) reaction experiments were performed. HACE1 (1:500 dilution, ab133637) was added after stripping the blot. The blot was observed after ECL color reaction.

### Flow cytometry

Cells (5×10^5^ cells) were collected and trypsinized without EDTA, after which they were resuspended in 500 μL of binding buffer. Annexin V-fluorescein isothiocyanate and propidium iodide (5 μL; Sanjiang Biological Technology Co., Ltd., China) were used to mark apoptotic cells, according to the manufacturer's instructions. Within 30 min, flow cytometry (BD FACSCalibur, USA) was used for detection.

### Transwell migration assay

Cells (5×10^4^ cells/mL) were seeded and cultured in a serum-free medium at 37 °C for 24 h for starvation treatment. After digestion, 100-μL cell solution (5×10^4^ cells/mL) was added to the hydrated Transwell chamber. After 24 h, unadhered cells were washed away. The cells that had infiltrated and migrated into the lower chamber were fixed with 95% ethanol and stained with 0.1% crystal violet. The number of migrated cells was counted in five random fields in a ×400-fold field of view.

### Co-immunoprecipitation

Cells were washed thrice with PBS and then lysed on ice. The lysate was centrifuged at 1200 rpm for 15 min to remove the precipitate. The supernatant was divided into three groups (positive control, negative control, and experimental group): the positive control was directly frozen for use; IgG was added to the negative control, and IP antibody was added to the experimental group (TRIP12, ab86220; HACE1, #730103; Thermo Fisher Scientific, USA), and then incubated overnight at 4°C. Then, 40-μL magnetic beads (Thermo Fisher Scientific) were added to each group, and after shaking at 4 °C for 1 h, the magnetic beads were washed five times with precooled PBS, after which protein loading buffer was added, and the target protein was detected using western blotting.

### Immunofluorescence assay

In the immunofluorescence (IF) assay, glass slides containing the cell climbing sheet were soaked in PBS (for 3 min × 3 times). The slides were fixed with 4% paraformaldehyde for 15 min, followed by soaking in PBS (for 3 min × 3 times). For permeabilization, cells were incubated with 0.5% Triton X-100 (in PBS) for 20 min at room temperature. After the slides were immersed in PBS (for 3 min × 3 times), normal goat serum was added dropwise to the slides and blocked at room temperature for 30 min. The blocking solution was sucked off using an absorbent paper, and a sufficient amount of rabbit polyclonal anti-HACE1 (1:50 dilution, PA5-83803; Thermo Fisher Scientific) or anti-TRIP12 (1:50 dilution, PA5-57840) was added to each slide and placed in a humid chamber, incubated at 4 °C overnight. The next day, after washing with Phosphate Buffered Saline with Tween 20 (PBST) (3 min × 3 times), goat antirabbit IgG H&L (1:500 dilution, ab150077; Alexa Fluor^®^ 488) was added, and the slides were incubated at 20-37 °C for 1 h in a humidified chamber. To counterstain the nucleus, DAPI was added, and the slides were incubated in the dark for 5 min, and excess DAPI was washed away with PBST (5 min × 4 times). After drying, the samples were mounted and then observed under a fluorescence microscope to capture images.

### Statistical analysis

All experiments were performed thrice independently. Data are expressed as mean ± standard deviation, and statistical analysis was performed using a one-way analysis of variance and Tukey's multiple comparison tests (GraphPad Prism, version 7.0). The t-test was used to analyze the differences between two groups. *P* < 0.05 indicated statistical significance.

## Results

### HACE1 expression is downregulated in ESCA tissues

**Figure 1:**
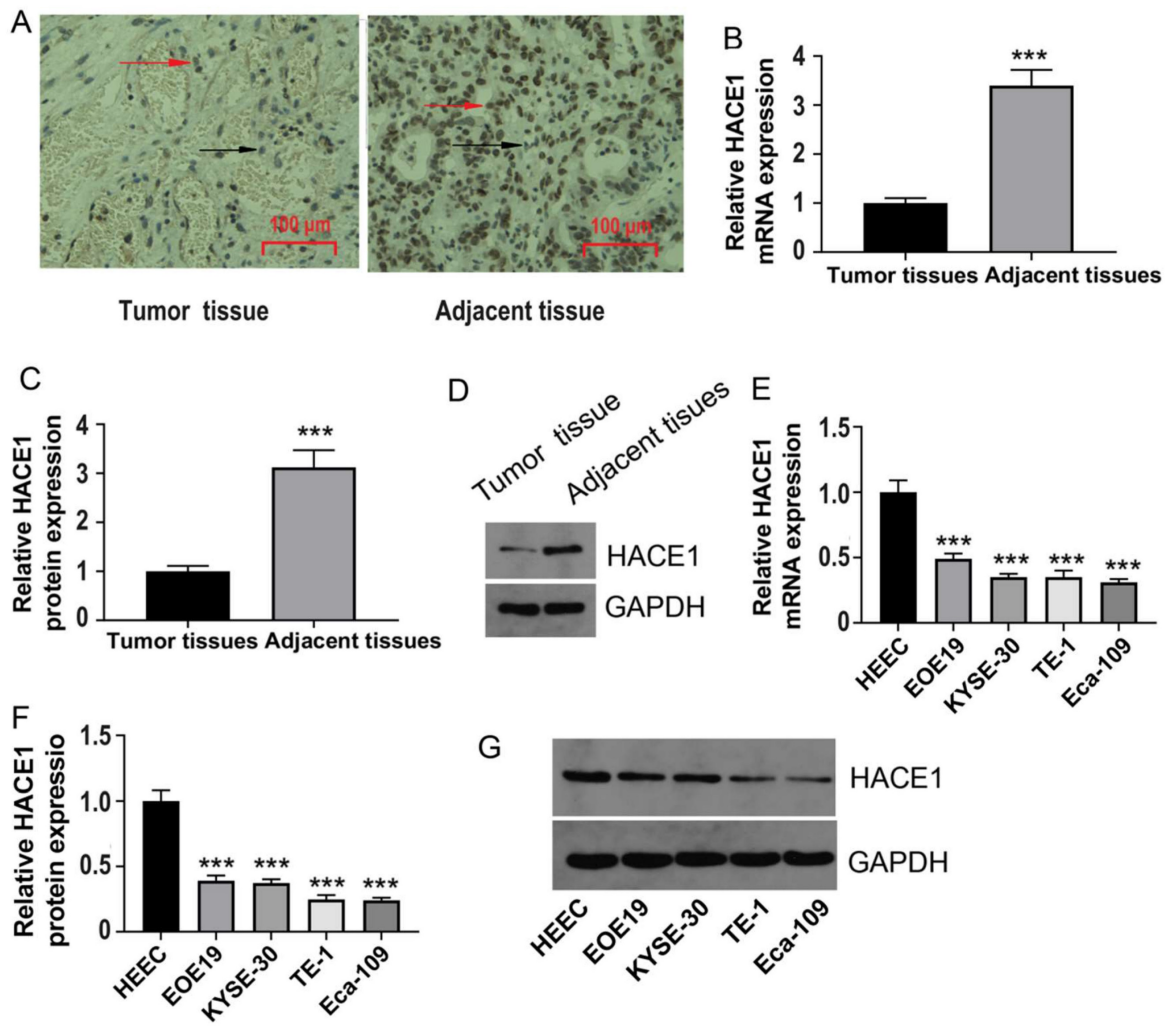
** HACE1 was downregulated in esophageal cancer (ESCA).** (A) Immunohistochemical staining to characterize HACE1 expression in ESCA tissues. The IHC-positive cells are indicated with red arrows; The IHC- negative cells are indicated with black arrows. (B-D) Comparison of HACE1 mRNA and protein expression in ESCA tissues and adjacent healthy tissues. (E-G) Comparison of HACE1 mRNA and protein expression in healthy human esophageal epithelial cells (HEEC) and ESCA cell lines. ^***^P < 0.001 vs. tumor tissues or HEEC.

To gain preliminary insights into the shifting trends of HACE1 within ESCA, we commenced by collecting clinical tissues for immunohistochemistry analysis. The outcomes unveiled reduced HACE1 levels in ESCA tissues juxtaposed with abundant HACE1 protein expression in normal tissues (Figure 1A). Moreover, quantitative experimental data further corroborated this observation, showcasing lowered HACE1 mRNA and protein levels in ESCA tissues relative to normal tissues (Figure 1B-1D). This pattern persisted in ESCA cell lines, as the levels of HACE1 mRNA and protein were notably downregulated when compared with normal esophageal epithelial cells (Figure 1E-1G). Accumulating evidence indicates a negative role for HACE1 in ESCA. Subsequently, we selected Eca-109 cells characterized by relatively low HACE1 expression and TE-1 cells marked by relatively high HACE1 expression for subsequent investigations.

### Overexpression of HACE1 inhibits the malignant biological behavior of ESCA cells

**Figure 2:**
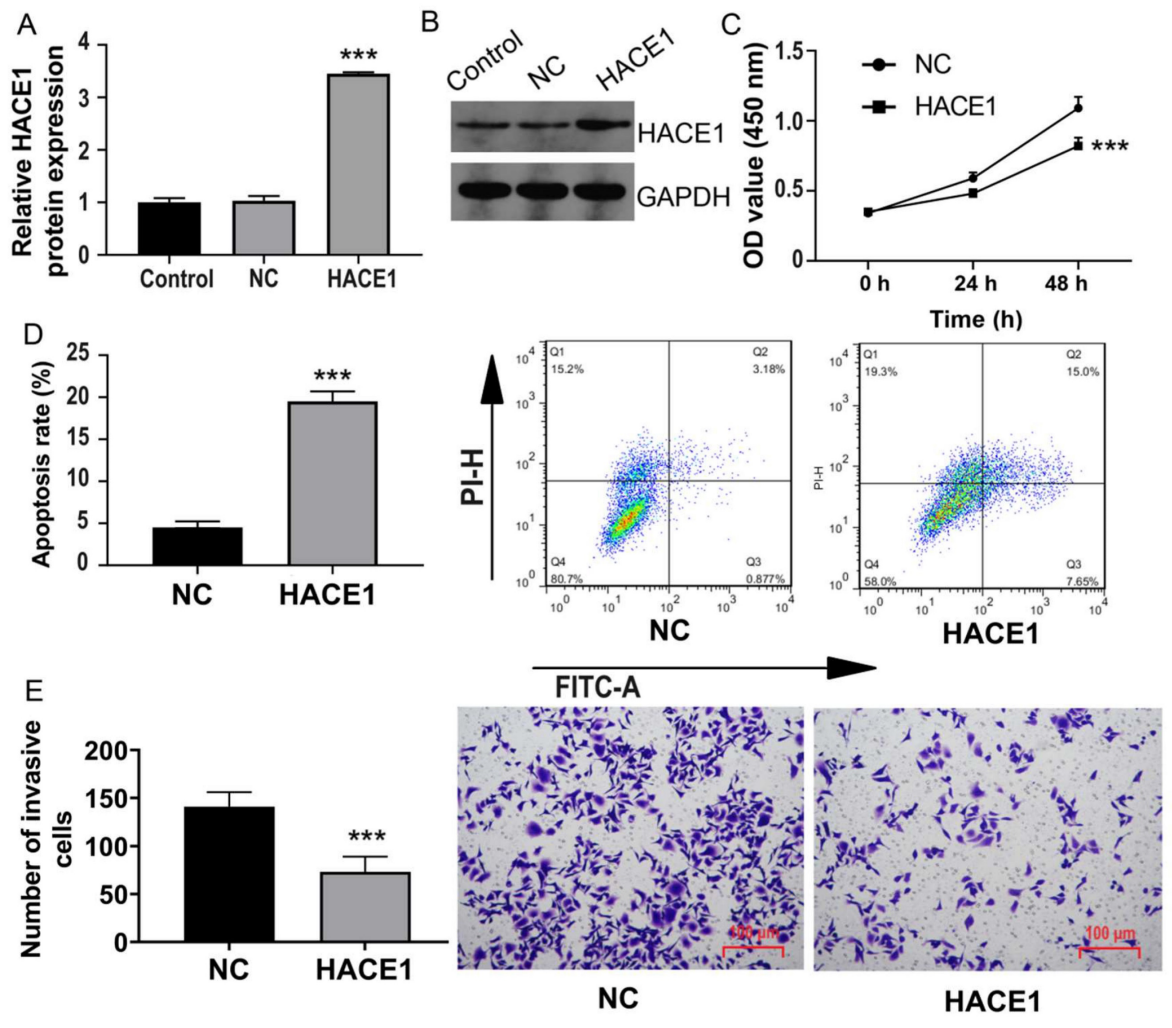
** HACE1 overexpression inhibited the malignant biological behavior of esophageal cancer (ESCA) cells.** (A-B) Comparison of the transfection efficiency of HACE1 overexpression in Eca-109 cells. (C) Effects of HACE1 overexpression on cell viability. (D) Effects of HACE1 overexpression on apoptosis. (E) Effects of HACE1 overexpression on invasion.^ ***^P < 0.001 vs. control or negative control (NC).

Among these ESCA cells, we chose Eca-109 cells with reduced HACE1 expression for transfection and overexpression of HACE1 (Figure 2A-2B). Notably, cell viability significantly reduced after HACE1 overexpression (Figure 2C), accompanied by a surge in the apoptosis rate from approximately 5% to approximately 20% (Figure 2D). Furthermore, the Transwell assay revealed diminished cell invasion in the HACE1 overexpression group in contrast to the negative control (NC) group (Figure 2E). Collectively, these findings underscore that HACE1 overexpression counteracts malignant biological behavior in ESCA cells.

### Silencing of HACE1 promotes ESCA cells

**Figure 3:**
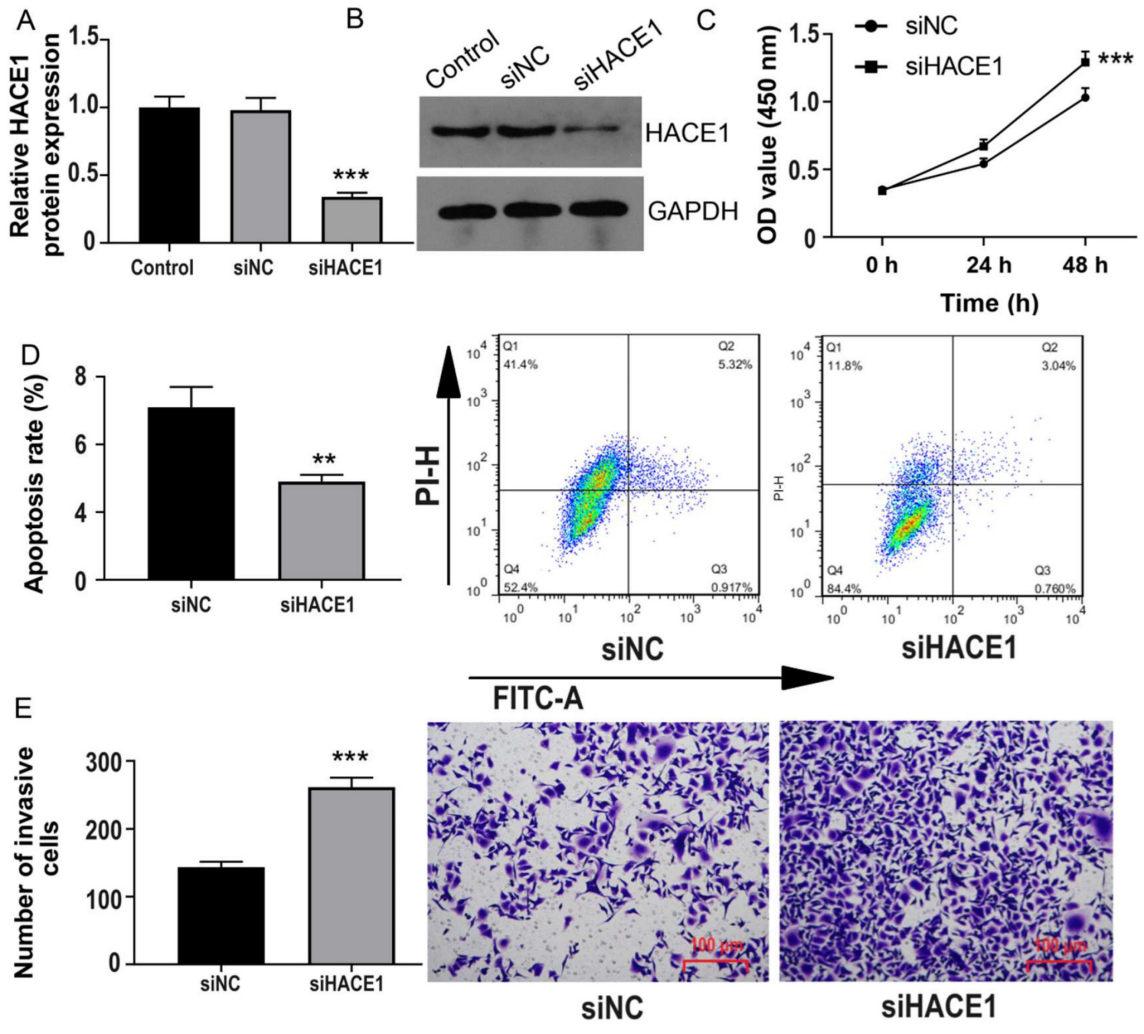
** HACE1 silencing promoted esophageal cancer (ESCA).** (A-B) Comparison of the transfection efficiency of HACE1 silencing in TE-1 cells. (C) Effects of HACE1 silencing on cell viability. (D) Effects of downregulated expression of HACE1 on apoptosis. (E) Effects of HACE1 silencing on tumor cell invasion.^ ***^P < 0.001 vs. control or siRNA negative control (siNC); **P < 0.01 vs. control or siRNA negative control (siNC).

Conversely, we turned our attention to TE-1 cells possessing elevated HACE1 expression and subjected them to HACE1 silencing (Figure 3A-3B). As HACE1 levels dwindled, cell proliferation escalated (Figure 3C). The inhibition of HACE1 moderately reduced the apoptosis rate from approximately 5% to approximately 3% (Figure 3D). Furthermore, the number of cells invading the lower chamber in the HACE1-silenced (siHACE1) group surpassed that in the siNC group (Figure 3E). These outcomes indicate that the suppression of HACE1 augments malignant biological tendencies in ESCA cells.

### HACE1 regulates the ubiquitination and degradation of RAC1 protein in ESCA cells

**Figure 4:**
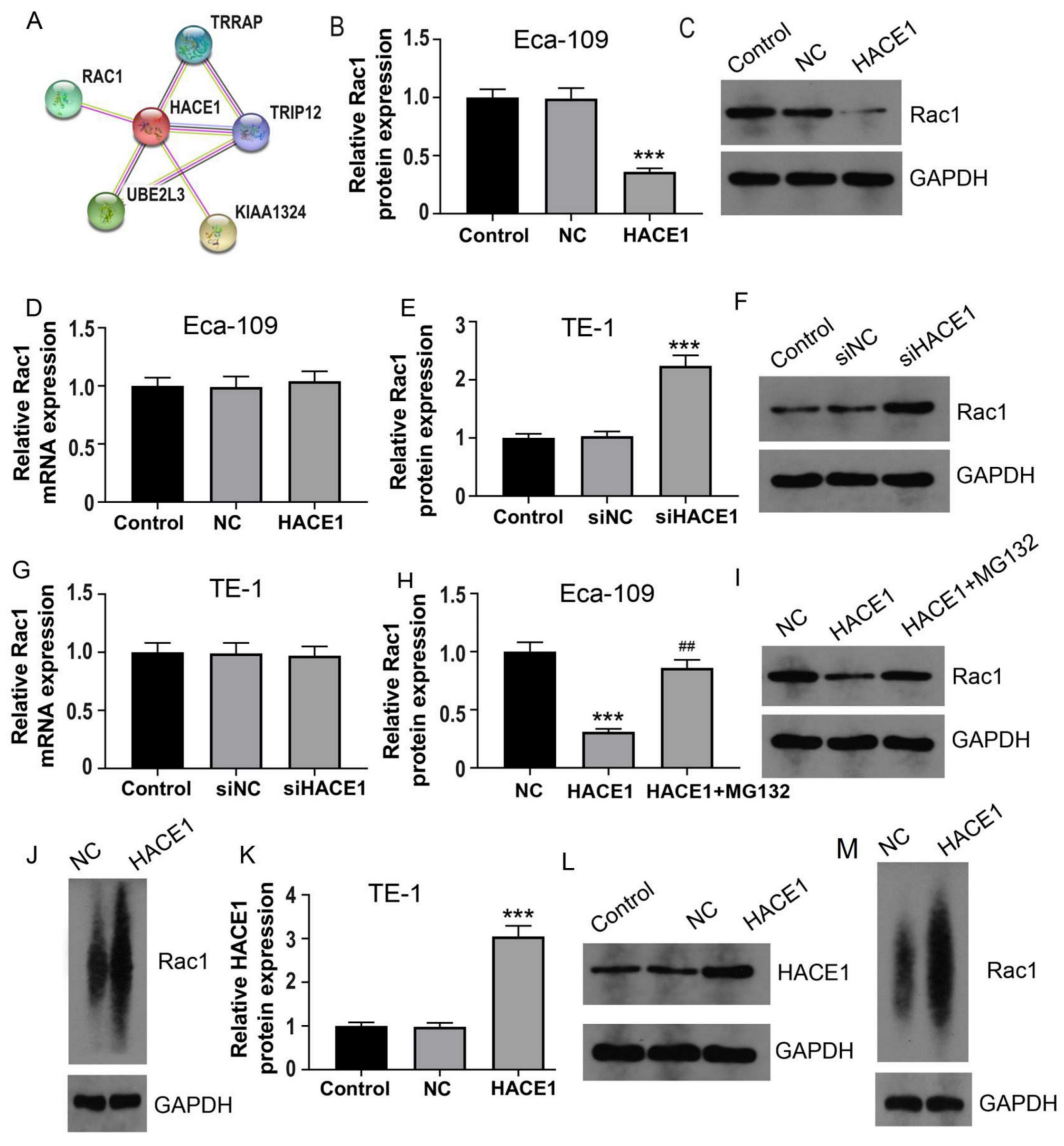
** HACE1 regulated the ubiquitination and degradation of RAC1 protein in esophageal cancer (ESCA) cells.** (A) Protein-protein interaction with HACE1 was analyzed using data from the String website. (B-D) Effects of HACE1 overexpression on RAC1 mRNA and protein expression levels in Eca-1 cells. (E-G) Effects of HACE1 silencing on RAC1 mRNA and protein expression levels in TE-1 cells. (H-I) Inhibitory effect of the proteasome inhibitor MG132 on HACE1-mediated RAC1 protein. (J) Effects of HACE1 on RAC1 protein ubiquitination in Eca-109. (K-L) Comparison of transfection efficiency of HACE1 overexpression in TE-1 cells. (M) Effects of HACE1 on RAC1 protein ubiquitination in TE-1 cells. ^***^P < 0.001 vs. control, negative control (NC) or siNC.

To delve into the molecular intricacies of HACE1's regulation of ESCA, we leveraged the String website for Protein-Protein Interaction (PPI) analysis. Figure 4A reveals five proteins closely associated with HACE1: RAC1, TRIP12, TRRAP, UBE2L3, and KIAA1324. As mentioned in the introduction, RAC1 elevation contributes to ESCA progression, prompting our initial investigation into the effects of HACE1 on RAC1. Intriguingly, HACE1 overexpression manifested no discernible impact on RAC1 mRNA levels but notably repressed RAC1 protein expression (Figure 4B-4D). Similarly, HACE1 silencing didn't influence RAC1 transcript levels but significantly heightened RAC1 protein levels (Figure 4E-4G). Given HACE1's status as an E3 ubiquitin ligase, we proceeded to examine RAC1 ubiquitination. The results unveiled that HACE1 overexpression substantially increased RAC1 protein ubiquitination in Eca-109 cells. Furthermore, when the proteasome system's ubiquitination degradation was impeded by MG132, HACE1's inhibition of RAC1 protein was also notably hindered (Figure 4H-4J). These findings were echoed in TE-1 cells, where augmented HACE1 levels correlated with increased RAC1 protein ubiquitination (Figure 4K-4M). This lends credence to the notion that HACE1's restraint on ESCA could be attributed to its ability to induce RAC1 protein ubiquitination and degradation.

### HACE1 inhibits tumor growth of ESCA by regulating RAC1

**Figure 5:**
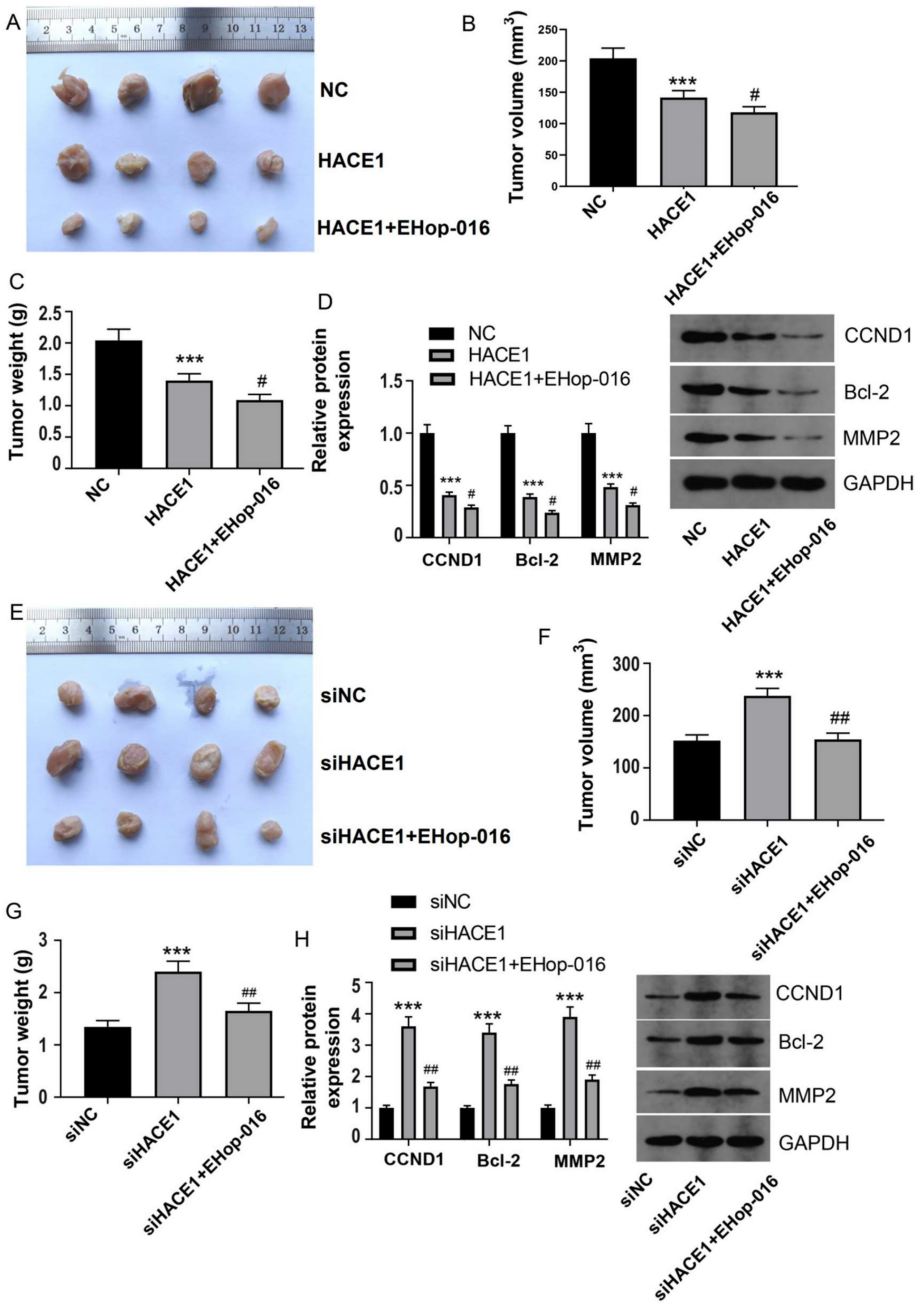
** HACE1 inhibited tumor growth of esophageal cancer (ESCA) by regulating RAC1.** (A-C) Effects of HAEC1 overexpression and the RAC1 inhibitor Ehop-016 on tumor growth in a tumor-bearing nude mouse model. (D) Effects of HAEC1 overexpression and the RAC1 inhibitor Ehop-016 on the protein expression of CCND1, Bcl-2, and MMP2 in tumor tissues. (E-G) Effects of HAEC1 overexpression and the RAC1 inhibitor Ehop-016 on tumor growth in the tumor-bearing nude mouse model. (H) Effects of HAEC1 overexpression and the RAC1 inhibitor Ehop-016 on the protein expression of CCND1, Bcl-2, and MMP2 in tumor tissues.^ ***^P < 0.001 vs. negative control (NC) or siNC; ^#^*P* < 0.05 vs. HACE1 and siHACE1; ^##^*P* < 0.01 vs. HACE1 and siHACE1.

To ascertain the in vivo implications of HACE1 inhibition of ESCA and the potential involvement of RAC1, we conducted tumor-bearing nude mouse experiments, employing a RAC1 inhibitor to block the RAC1 pathway. Employing Eca-109 cells overexpressing HACE1, the resulting model mirrored cell experiment outcomes, with tumor volume and mass significantly suppressed upon HACE1 elevation (Figure 5A-5C). Subsequent inhibition of the RAC1 pathway by EHop-016 further reduced tumor volume and mass (Figure 5A-5C). In concordance, HACE1 suppressed the expression of CCND1, Bcl-2, and MMP2 in tumor tissue, with EHop-016 intensifying the inhibitory effects on these proteins (Figure 5D). The model constructed with HACE1-silenced TE-1 cells exhibited heightened tumor volume and mass post-HACE1 inhibition (Figure 5E-5G). However, tumor growth was restored when the RAC1 pathway was inhibited by EHop-016 (Figure 5E-5G). Notably, the levels of CCND1, Bcl-2, and MMP2 were elevated in the siHACE1 group compared to the NC group, but these levels were notably lower in the siHACE1+EHop-016 group relative to the siHACE1 group (Figure 5H). These results collectively underscore that inhibiting RAC1 nullifies the promotion of ESCA prompted by HACE1 downregulation, affirming the integral role of RAC1 in HACE1's inhibition of ESCA.

### HACE1 regulates the ubiquitination degradation of RAC1 protein via TRIP12 in Eca-109

**Figure 6:**
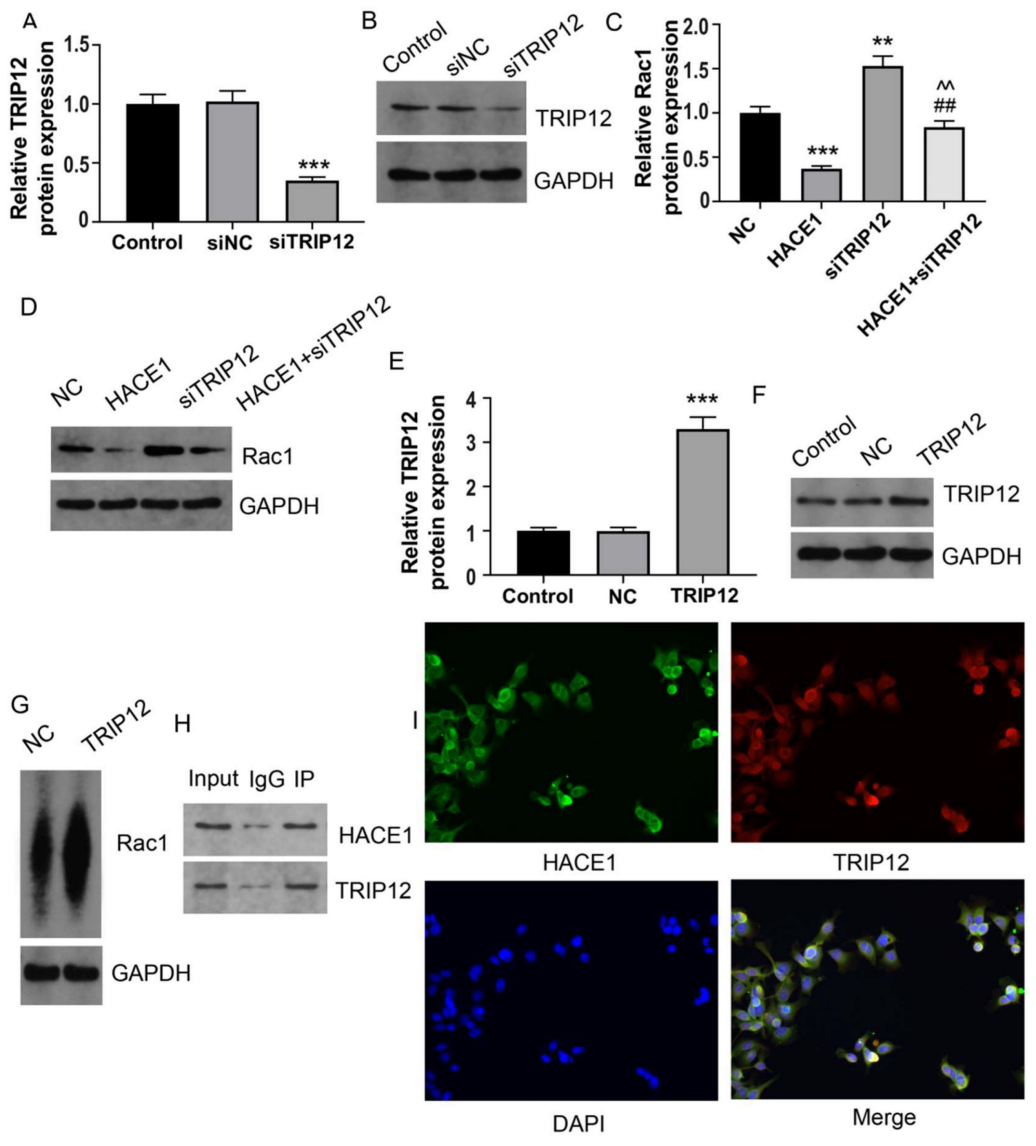
** HACE1 regulated the ubiquitination degradation of the RAC1 protein through TRIP12 in Eca-109.** (A-B) Comparison of the transfection efficiency of TRIP12 silencing in Eca-109 cells. (C-D) Effects of HACE1 overexpression and/or TRIP12 silencing on RAC1 protein expression levels. (E-F) Comparison of transfection efficiency of TRIP12 overexpression. (G) Effects of TRIP12 overexpression on RAC1 protein ubiquitination. (H) Co-IP validation of the protein-protein interaction between HACE1 and TRIP12. (I) Immunofluorescence detection of the subcellular localization of HACE1 and TRIP12. ^**^P < 0.01, ^***^P < 0.001 vs. control, negative control (NC) or siNC; ^##^*P* < 0.01 vs. HACE1; ^^^^*P* < 0.01 vs. siTRIP12.

Based on the predictive outcomes of HACE1, it came to light that, TRIP12 also acted as an E3 ubiquitin ligase. This observation prompted the speculation that TRIP12 might play a role in mediating RAC1 ubiquitination under the influence of HACE1. There have been some studies on the relationship between RAC1 and E3 ubiquitin ligases. Ma et al showed that the E3 ubiquitin ligase MG53 acted as a direct inhibitor of RAC1 18. Another study demonstrated that the E3 ubiquitin-ligase HACE1 catalyzed the ubiquitylation of active Rac1 19. It's also shown that the ubiquitin E3 ligase TRAF6 exacerbates ischemic stroke by ubiquitinating and activating RAC1 20. TRIP12 has been shown to be an E3 ubiquitin ligase in many previous studies 21-24. To tentatively ascertain TRIP12's involvement, we generated Eca-109 cells with silenced TRIP12 expression (Figure 6A-6B). The Eca-109 cells were then categorized into four groups: NC, HACE1, siTRIP12, and HACE1+siTRIP12. A comparative analysis demonstrated that the RAC1 protein levels in the HACE1 group exhibited reduction, while those in the siTRIP12 group displayed elevation relative to the NC group. Interestingly, the RAC1 protein levels in the HACE1+siTRIP12 group were higher than those in the HACE1 group, yet markedly lower than those in the siTRIP12 group (Figure 6C-6D). By effectively silencing TRIP12, the inhibitory effects of HACE1 on RAC1 were effectively nullified. To corroborate the effect of TRIP12 on RAC1 ubiquitination, we constructed Eca-109 cells overexpressing TRIP12 (Figure 6E-6F), which led to an evident elevation in the ubiquitination level of RAC1 (Figure 6G). Co-IP experiments provided validation of the cellular interaction between HACE1 and TRIP12 (Figure 6H). Immunofluorescence (IF) detection further affirmed the co-localization of TRIP12 and HACE1 within Eca-109 cells (Figure 6I). Collectively, these observations point toward a close interrelation between the process of HACE1-induced RAC1 ubiquitination and TRIP12.

### HACE1 regulates the cell biological behavior of ESCA through TRIP12

**Figure 7:**
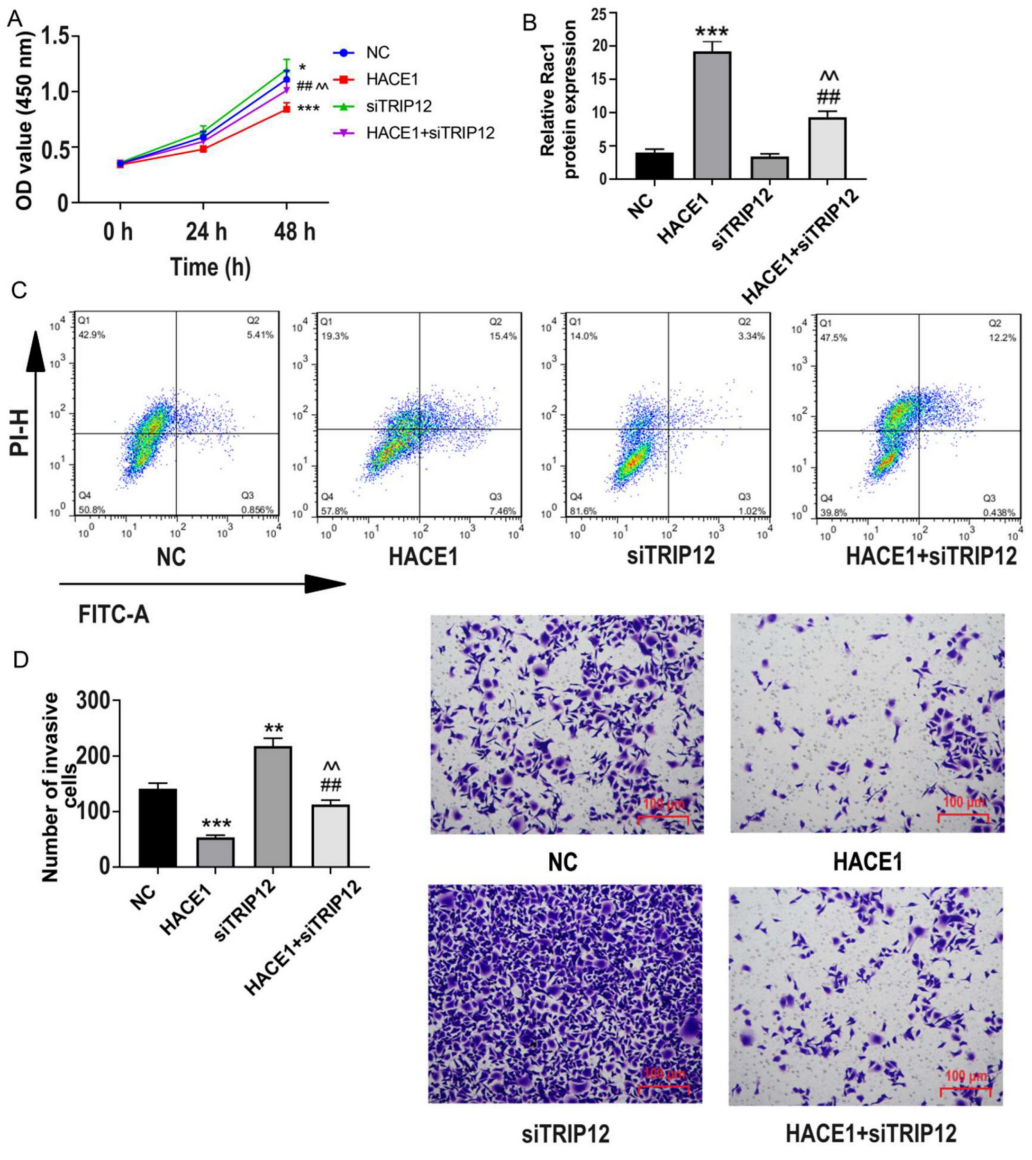
** HACE1 regulated the biological behavior of esophageal cancer (ESCA) cells through TRIP12.** (A) Effects of HACE1 overexpression and/or TRIP12 silencing on cell viability. (B-C) Effects of HACE1 overexpression and/or TRIP12 silencing on apoptosis. (D) Effects of HACE1 overexpression and/or TRIP12 silencing on invasion.^ *^P < 0.05, **P < 0.05, ^**^P < 0.001 vs. negative control (NC); ^##^*P* < 0.01 vs. HACE1; ^^^^*P* < 0.01 vs. siTRIP12.

Further probing the impact of TRIP12 in HACE1-related ESCA dynamics, we investigated the biological behavior of four distinct groups: NC, HACE1, siTRIP12, and HACE1+siTRIP12. Although TRIP12 silencing marginally boosted cell viability, it notably offset HACE1's inhibitory effects on Eca-109 ESCA cells (Figure 7A). A similar pattern emerged in terms of apoptosis, with the HACE1+siTRIP12 group exhibiting lower apoptosis rates relative to the HACE1 group and higher than those of the siTRIP12 group (Figure 7B-7C). Additionally, cell invasiveness in the HACE1+siTRIP12 group was higher than in the HACE1 group yet lower than the siTRIP2 group (Figure 7D). These results underscore that while TRIP12's promotion effects on ESCA cells are subtle, its silencing markedly neutralizes HACE1's inhibitory impact on ESCA, underscoring TRIP12 indispensable role in HACE1-mediated inhibition.

### Silencing of TRIP12 blocks the inhibition of HACE1 on ESCA tumor growth and RAC1 in vivo

**Figure 8:**
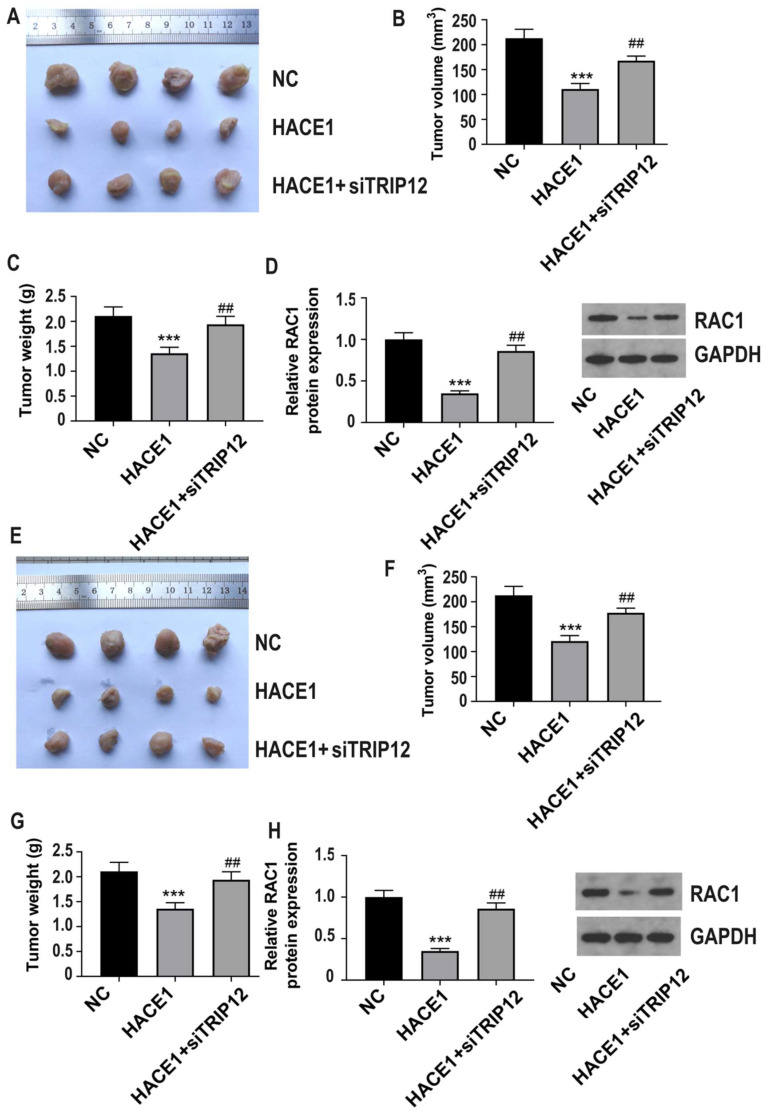
** TRIP12 silencing blocks the inhibitory effect of HACE1 on tumor growth in esophageal cancer (ESCA) and RAC1 in vivo.** (A-C) Effects of HACE1 overexpression and/or TRIP12 silencing on tumor growth in a tumor-bearing nude mouse model. (D) Effects of HACE1 overexpression and/or TRIP12 silencing.

For in vivo studies, we used Eca-109 cells in three groups: NC, HACE1, and HACE1+siTRIP12, and observed that TRIP12 silencing significantly exacerbated tumor growth on the basis of HACE1 overexpression (Figure 8A-8C). Furthermore, the RAC1 protein level was lower in the HACE1 group than the NC group, but elevated in the HACE1+siTRIP12 group (Figure 8D). Similar trends were observed using TE-1 cells in three groups: NC, HACE1, and HACE1+siTRIP12. Notably, the HACE1+siTRIP12 group exhibited significantly enhanced tumor growth compared to the HACE1 group (Figure 8E-8G). Additionally, TRIP12 silencing neutralized HACE1's inhibitory effects on RAC1 protein levels within tissues (Figure 8H). These in vivo results reinforce the assertion that HACE1's inhibition of ESCA progression and RAC1 protein expression hinges on TRIP12.

## Discussion

ESCA is one of the top ten cancers worldwide, with China experiencing notably high incidence rates. The country witnesses an annual average of approximately 150,000 ESCA-related deaths, predominantly affecting individuals over 40 years old 25-26. For individuals diagnosed with advanced ESCA, comprehensive treatments yield a disheartening 5-year survival rate of below 10% 27-28. A limited comprehension of ESCA's pathogenesis hampers the development of effective diagnostic and therapeutic strategies.

HACE1, a protein conserved across multiple species, assumes a pivotal role in preserving astrocyte mitochondrial function and ensuring normal nervous system development 29-30. Extensive evidence underscores HACE1's role as a tumor suppressor in various human cancers. For instance, elevated methylation levels of the HACE1 gene have been observed in liver cancer cells; conversely, demethylation of the HACE1 promoter significantly curbs proliferation 31. Furthermore, HACE1 demonstrates tumor-suppressive properties in Wilms' tumor 32, osteosarcoma 33, and colorectal cancer 34. However, a counterintuitive finding suggests that HACE1 might promote melanoma's lung metastasis 35. Within the realm of ESCA, the exact mechanisms governed by HACE1 remain obscure. This study initiates histological and cytological analyses, revealing a significant down-regulation of HACE1 in both ESCA tissues and cell lines. Subsequent investigations, employing cell culture and nude mouse models, unveil that diminished HACE1 expression enhances ESCA cell proliferation and invasiveness while inhibiting apoptosis, both in vivo and in vitro. Conversely, elevating HACE1 levels impede ESCA progression. Collectively, these findings corroborate HACE1's role as a tumor suppressor within the realm of ESCA.

To gain preliminary insights into the mechanism of HACE1's action, we conducted an analysis based on PPI. Our findings highlight the existence of PPI between HACE1 and RAC1. RAC1, a GTPase associated with the plasma membrane, becomes potent in its active state, driving the proliferative and metastatic tendencies of ESCA cells 36-37. Given RAC1's potential as a therapeutic target for ESCA, it's plausible that HACE1 might inhibit ESCA through its interaction with RAC1 38. Our study revealed that alterations in HACE1 levels had negligible effects on RAC1 mRNA expression, yet substantially impacted RAC1 protein levels. Since HACE1's influence on RAC1 appears to be at the protein level rather than transcriptional, and considering its role as an E3 ubiquitin ligase, there is a possible a preliminarily speculation that HACE1 might steer ESCA progression by initiating the ubiquitination and subsequent degradation of RAC1. This hypothesis was validated by subsequent experimental results. HACE1 increased the ubiquitination of RAC1 protein, and this effect was counteracted when the proteasome was inhibited by MG132. Animal experiments further substantiated this notion, demonstrating that EHop-016 countered the promoting effects of HACE1 silencing on ESCA by impeding RAC1 protein function. Prior reports suggesting HACE1-mediated RAC1 ubiquitination repressed the proliferation of breast and lung cancer cells further corroborate the notion that HACE1 suppresses ESCA progression through RAC1 ubiquitination 39-40.

To delve deeper into the molecular mechanism underlying HACE1's promoting effect of RAC1 ubiquitination, we explored other proteins that interact with HACE1 through PPI. Interestingly, TRIP12 emerged as another E3 ubiquitin ligase similar to HACE1 41-42. A study unveiled TRIP12's involvement in mediating the degradation of the tumor suppressor protein FBW7, influencing leukemia progression 43. The ability of TRIP12 to inhibit tumor growth in a lung cancer mouse model through CD8+ T cells 44 has also been documented. In breast cancer, TRIP12 inhibits epithelial-mesenchymal transition through the ubiquitin-mediated degradation of ZEB1/2 45. To ascertain TRIP12's role in regulating RAC1 through HACE1, cellular experiments were conducted. The results showcased that while TRIP12 silencing had minor effects on the behavior of ESCA cells, it remarkably counteracted HACE1's inhibitory effects on RAC1. Co-IP verified the existence of PPI between HACE1 and TRIP12 within cells, substantiating their shared cellular localization. Animal experiments concurred, displaying that TRIP12 silencing mitigated HACE1's inhibitory impact on tumor growth and RAC1 protein levels in tumor tissue. This lends credence to the notion that HACE1's promotion of RAC1 ubiquitination is intricately tied to TRIP12. Thus, HACE1 propels RAC1 protein ubiquitination by binding with TRIP12.

However, there are some limitations in the study that need to be discussed. Due to the importance of HACE1 in ESCA and its potential implications, there is a pressing need for extensive research that involves clinical samples. Moreover, the binding mode of HACE1 and TRIP12, as well as the intricate mechanism governing RAC1 ubiquitination, requires thorough investigation to ensure accuracy and reliability of the findings. Therefore, additional verification becomes essential in order to advance our understanding in this field and pave the way for potential therapeutic applications.

## Conclusions

Our findings illuminate that HACE1, downregulated in ESCA, curbs ESCA progression by triggering the ubiquitinated degradation of RAC1 protein via its interaction with TRIP12. To our best knowledge, this is the first study to show that the expression of HACE1 was significantly decreased in the ESCA tissues and HACE1 inhibited the malignant biological behavior of ESCA. Our study also firstly confirmed that TRIP12 was an important factor in the esophageal cancer progression. Most importantly, TRIP12 inhibition blocked HACE1-driven RAC1 ubiquitination and mitigated the inhibitory effects of HACE1 on ESCA cells, alleviating tumor growth in the tumor-bearing nude mouse model. These results may provide insights into the development of medicine against ESCA in the future. This underscores the potential of TRIP12, HACE1 and RAC1 as novel targets for ESCA diagnosis and treatment, which may help developing new medicines for ESCA.
